# The complete mitochondrial genome of *Ancherythroculter nigrocauda* (Cypriniformes, Cyprinidae) and its phylogenetic position

**DOI:** 10.1080/23802359.2020.1787902

**Published:** 2020-07-11

**Authors:** Ping Li, Huamei Pan, Jinjin Wang

**Affiliations:** a College of Animal Science, Key Laboratory of Freshwater Fish Reproduction and Development (Ministry of Education), Southwest University, Chongqing, China; b School of Life Sciences, Southwest University, Chongqing, China

**Keywords:** Mitochondrial DNA, *Ancherythroculter nigrocauda*, Oxygastrinae, phylogeny

## Abstract

The complete mitochondrial genome of *Ancherythroculter nigrocauda* was determined in this study. It contained 1 replication origin, 1 control region (D-Loop), 2 rRNA genes, 13 PCGs, and 22 tRNA genes with the base composition 31.40% A, 25.00% T, 27.60% C, and 16.00% G. Here, we compared this newly determined mitogenome with another one from the same species reported before. The variable sites and the genetic distances between the two mitogenomes were 608 bp and 0.038, respectively. The results from the phylogenetic analysis showed that the genus *Ancherythroculter* is not a monophyletic group and *Ancherythroculter nigrocauda* demonstrates a close relationship with *Chanodichthys dabryi*.

*Ancherythroculter nigrocauda* (Cypriniformes, Cyprinidae, Oxygastrinae) is an endemic species in China, distributed in Sichuan and Chongqing (Froese and Pauly 2019). It has high economic value and nutritional value. Here, the complete mitochondrial genome sequence of *A*. *nigrocauda* was determined (GenBank accession No. MT588183) and was compared with another *A*. *nigrocauda* mitogenome data reported before (Wan et al. 2013). The specimens (voucher no. SWU20110226005) were collected from Jialing River (29.97 N, 106.28 E), Hechuan, Chongqing, China, and were stored in the museum of the Key Laboratory of Freshwater Fish Reproduction and Development (Ministry of Education, Southwest University, Chongqing, China). We extracted DNA and designed primers according to the method of Wang et al. (2011). The mitochondrial genome is annotated via the MITOS WebServer (Bernt et al. 2013).

The complete mitochondrial genome of *A*. *nigrocauda* was a circular molecule with 16,620 bp in length. It contained 1 replication origin, 1 control region (D-Loop), 2 rRNA genes, 13 PCGs, and 22 tRNA genes. Most of the genes were encoded on H-strand, while *ND6* and 8 tRNA genes (tRNA-Gln, tRNA-Ala, tRNA-Asn, tRNA-Cys, tRNA-Tyr, tRNA-Ser, tRNA-Glu, and tRNA-Pro) were encoded on L-strand. The overall nucleotide composition was 31.40% A, 25.00% T, 27.60% C, and 16.00% G, with a slight AT bias. All the mitochondrial PCGs in the *A*. *nigrocauda* use the standard ATG start codon, except for *CO1*, which utilizes GTG. Six PCGs (*ND1*, COI, *ATP6*, *CO III*, *ND4L*, and *ND5*) contain TAA stop codon, five PCGs (*ND2*, *ATP8*, *ND3*, *ND4*, and *ND6*) contain TAG stop codon and two PCGs (*COII*, *Cytb*) contain the incomplete stop codon T–. The arrangement of complete mitochondrial genome of *A*. *nigrocauda* is basically the same as the previous research (Li et al. 2012; Wan et al. 2013).

Comparing with another mitogenome of *A*. *nigrocauda*, the length of it was 16,623 bp (accession no. KC513573; Wan et al. 2013). The genetic distance between the two mitogenomes was 0.038. Two indels and 608 variable sites exist in between the two mitogenomes. Among these variable sites, 566 transitions (93%) and 42 transversions (7%) were found.

To confirm the phylogenetic position of *A*. *nigrocauda* among the genus *Ancherythroculter*, phylogenetic analysis based on the complete mitogenome using maximum-likelihood was conducted using IQ-tree (Nguyen et al. 2015; Hoang et al. 2018). The ModelFinder was used to calculate the optimal nucleotide substitution model (Kalyaanamoorthy et al. 2017). Phylogenetic maximum likelihood tree is given (Figure 1). The results from the analyses show that the genus *Ancherythroculter* is not a monophyletic group and *A*. *nigrocauda* demonstrates a close relationship with *Chanodichthys dabryi*.

**Figure 1.**
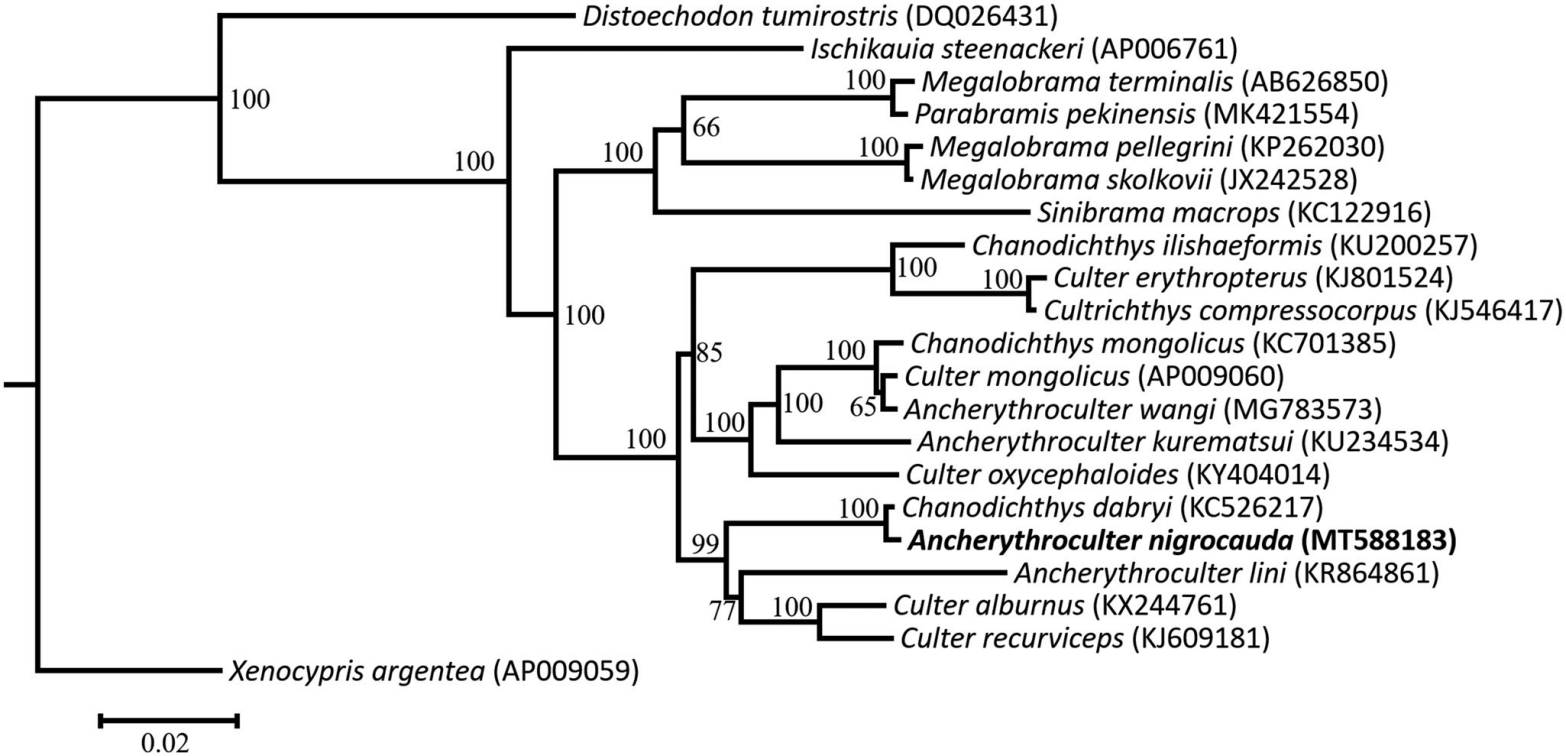
Phylogenetic tree of *Ancherythroculter nigrocauda* and the other 19 Cyprinidae species based on maximum likelihood (ML) method. The *Xenocypris argentea* is used as an outgroup. The ML bootstrap value is shown at the nodes.
